# Supplementary material for: Targeted Delivery of Nanoparticulate Cytochrome C into Glioma Cells Through the Proton-Coupled Folate Transporter
Source: Biomolecules. 2019 Apr 18;9(4):154. doi: 10.3390/biom9040154 (PMC6523331; doi:10.3390/biom9040154)
Supplement: Supplementary file 1 [file biomolecules-09-00154-s001.pdf]

Supplementary Materials

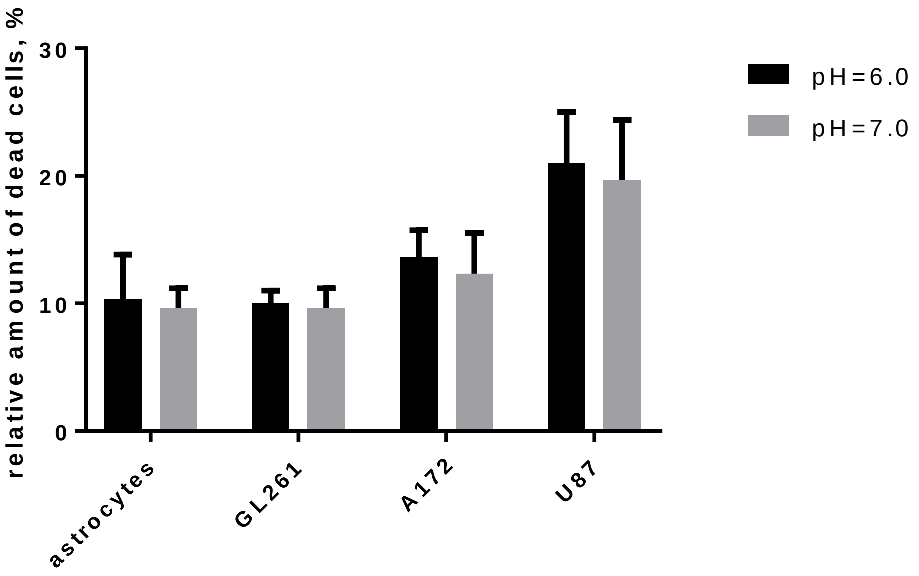

Figure S1. The viability of astrocytes and glioma cells, maintained in an acidic (pH = 6.0) and neutral (pH = 7.0) cell culturing medium for 24 hours. Live and dead cells were counted with the use of trypan blue staining. The proportion of dead cells was evaluated as the percentage of the total number of cells. Mean ± S.E. are shown. $N = 5$.

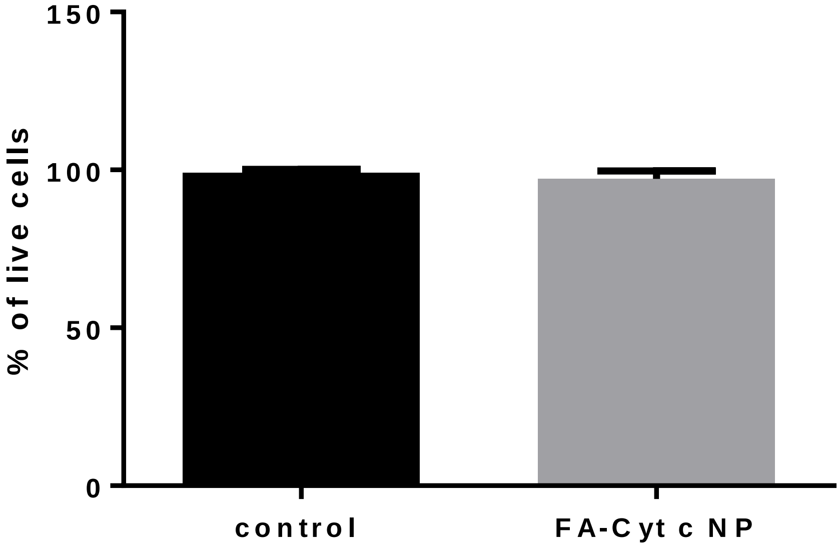

Figure S2. The 5-day viability of mouse primary cultured astrocytes treated with FA-coated Cyt c NPs (100 μg/ml). Live/dead assay based on calcein (green) and ethidium homodimer-1 (red) staining of live and dead cells was performed after 5 days of treatment with FA-Cyt c NPs. The number of dead cells as a percentage of the total number of cells, mean ± S.E is shown. $N = 3$.

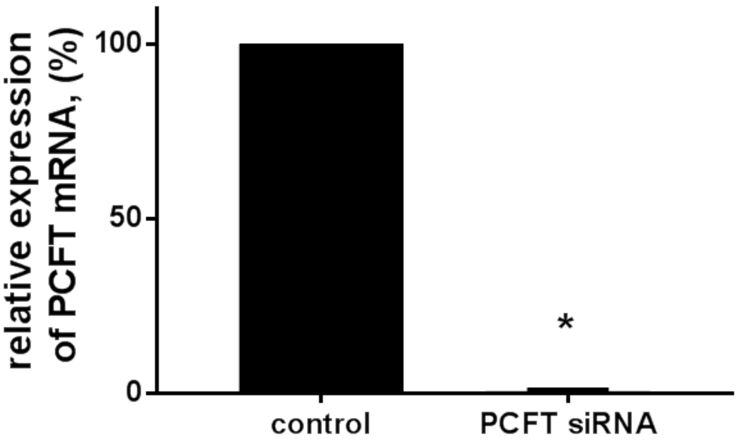

Figure S3. Relative gene expression of PCFT for control (Mock transfected) GL261 glioma cells and cells transfected with 10 nM siRNA against PCFT (SLC46A1). Data are normalized to Glyceraldehyde 3-phosphate dehydrogenase (GAPDH) as housekeeping gene. Mean ± SEM, significant differences from control (*) are shown ($p < 0.05$). $N = 3$.

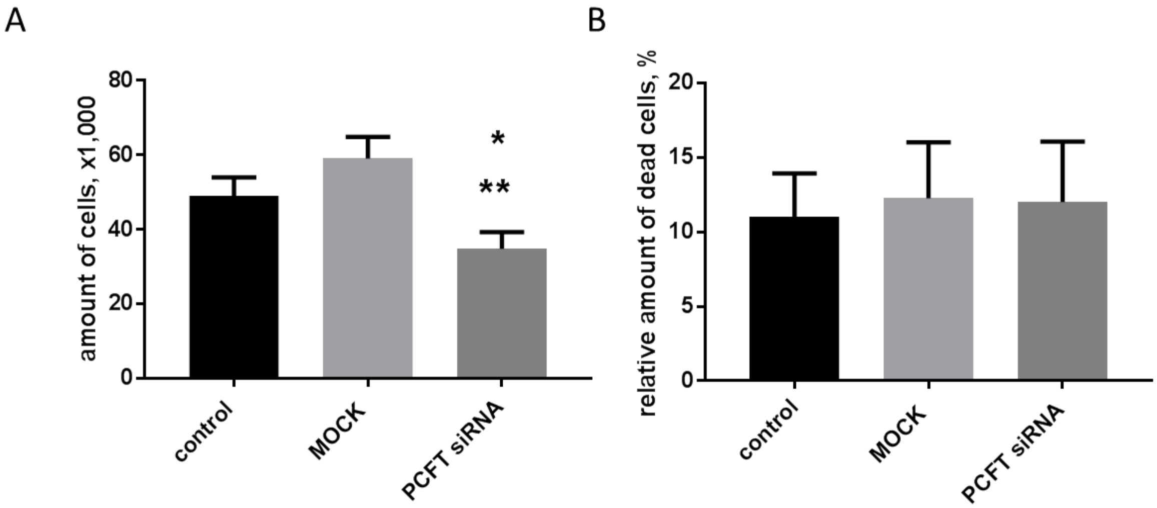

Figure S4. The viability of GL261 glioma cells transfected or un-transfected with siRNA against the PCFT. Live and dead cells were counted with the use of trypan blue staining. (A) Cell viability was evaluated as the total number of live cells. (B) The proportion of dead cells was evaluated as the percentage of the total number of cells. Mean ± S.E.; significant differences from control (*) and from MOCK (**) are shown ($p < 0.05$). $N = 5$.
